# Performance Study of Ultraviolet AlGaN/GaN Light-Emitting Diodes Based on Superlattice Tunneling Junction

**DOI:** 10.3390/mi16010028

**Published:** 2024-12-28

**Authors:** Zhuang Zhao, Yang Liu, Peixian Li, Xiaowei Zhou, Bo Yang, Yingru Xiang, Junchun Bai

**Affiliations:** 1 School of Advanced Materials and Nanotechnology, Xidian University, Xi’an 710071, China; 22141214314@stu.xidian.edu.cn (Z.Z.); yliu_333@stu.xidian.edu.cn (Y.L.); yangbo1@stu.xidian.edu.cn (B.Y.); 22141214236@stu.xidian.edu.cn (Y.X.); 2 State Key Discipline Laboratory of Wide Band Gap Semiconductor Technology, Xidian University, Xi’an 710071, China; 23113110590@stu.xidian.edu.cn

**Keywords:** ultraviolet light-emitting diodes, tunnel junction, APSYS

## Abstract

In this study, we aim to enhance the internal quantum efficiency (IQE) of AlGaN-based ultraviolet (UV) light-emitting diodes (LEDs) by using the short-period AlGaN/GaN superlattice as a tunnel junction (TJ) to construct polarized structures. We analyze in detail the effect of this polarized TJ on the carrier injection efficiency and investigate the increase in hole and electron density caused by the formation of 2D hole gas (2DHG) and 2D electron gas (2DEG) in the superlattice structure. In addition, a dielectric layer is introduced to evaluate the effect of stress changes on the tunneling probability and current spread in TJ. At a current of 140 mA, this method demonstrates effective current expansion. Our results not only improve the performance of UV LEDs but also provide an important theoretical and experimental basis for future research on UV LEDs based on superlattice TJ. In addition, our study also highlights the key role of group III nitride materials in achieving efficient UV luminescence, and the polarization characteristics and band structure of these materials are critical for optimizing carrier injection and recombination processes.

## 1. Introduction

In recent years, AlGaN-based ultraviolet (UV) light-emitting diodes have gained attention due to several advantages, including compact size, low power consumption, tunable wavelength, eco-friendliness, and extended lifespan [1,2]. These characteristics have led to their widespread use in various sectors, such as sterilization and disinfection, sensing, water purification, medical applications, and non-line-of-sight communication [3]. However, despite ongoing advancements in material and device fabrication technologies, AlGaN-based deep-ultraviolet LEDs still face several challenges, including limited confinement capability in multiple quantum wells (MQWs), inefficient p-type doping in high-Al-content AlGaN, and the polarization field-induced quantum-confined Stark effect (QCSE) [4,5]. These issues result in significant electron leakage, inadequate hole injection, and low radiative recombination efficiency, which hinder the advancement of UV LEDs [6,7]. Therefore, research on methods to enhance the efficiency of UV LEDs is essential for expanding their applications across various fields. Improving the luminescence efficiency of AlGaN-based UV LEDs primarily focuses on increasing the internal quantum efficiency (IQE) and light extraction efficiency (LEE) [8]. As is well known, the external quantum efficiency (EQE) is determined by the IQE and LEE, where the IQE is determined by the material’s quality, the carrier injection, and recombination, and the LEE is determined by the total internal reflection (TIR), the optical absorption, and the optical polarization [9]. In order to improve the EQE, high-efficiency carrier injection is required to realize high-performance AlGaN-based UV LEDs. The injection efficiency of an LED, also referred to as carrier injection efficiency, is defined as the ratio of the current injected into the active region to the total current supplied to the LED. Factors influencing carrier injection efficiency in LEDs include electron and hole concentrations, electric field strength, and potential barrier height. Currently, methods to enhance carrier injection efficiency include structures such as superlattice p-type electron blocking layers (EBLs) [10], AlN insertion layer structures [11], multilayer EBL structures [12,13], polarization-modulated EBLs [14], tunnel junctions [15,16,17], hybrid polarization [18], conventional co-doping of p-regions [19], etc.

A tunnel junction (TJ) is a heavily doped p^+^−n^+^ junction with a very thin barrier, where the electron and hole concentrations typically exceed 1 × 10^20^ cm^−3^ and the thickness is approximately 10 nm. The tunnel effect usually refers to the physical phenomenon whereby an extremely thin insulating layer (thickness of about several nanometers, such as oxide film) exists between two metals. When potential energy is applied at both ends to form a barrier, some particles with kinetic energy in the conductor can pass the barrier from one side of the insulation layer to the other side, even under the conditions of the particle energy being less than the barrier height [20]. The following three conditions are usually required for efficient tunneling: (i) The Fermi level (E_F_) must be located within the conduction and valence of the materials from the opposite sides of the barrier, meaning that the energy levels of the electrons and holes must overlap or nearly overlap in order for the particle to have enough energy to cross the forbidden band. (ii) The space charge region of the tunneling junction (also known as the depletion region) must be very narrow, usually less than 10 nm. This is because the width of the space charge region determines the tunneling probability, and the narrower the width, the higher the tunneling probability of electrons and holes across the forbidden band. (iii) Very high doping concentrations, p^+^ and n^+^ doping, are usually required on both sides of the tunnel junction to ensure high concentrations of electrons and holes. This helps to achieve a degenerate semiconductor, which increases the tunneling current. High doping concentrations can increase the number of carriers, thereby increasing the chance of tunneling events. When an LED incorporates a built-in tunnel junction, the electrons in the valence band of the p-type semiconductor can pass through the gap band and enter the conduction band of the n-type semiconductor under the action of reverse bias, and the tunneling current increases monotonically with the reverse voltage. Electrons in the tunnel junction must cross the potential barrier created by the forbidden band during tunneling, generating relatively high tunneling resistance. This suppresses the current crowding effect of the LED and improves transverse current diffusion. The n-type layer also acts as a contact layer for the electrodes, facilitating ohmic contact.

**Figure 1:**
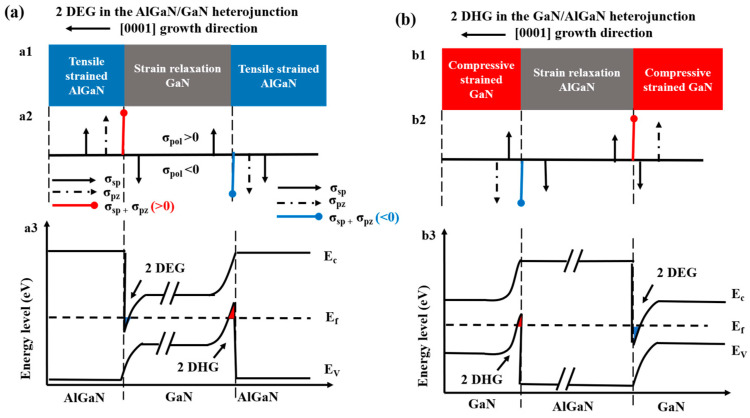
AlGaN/GaN heterojunction polarization induced the formation of 2DEG and 2DHG. a1 and b1 represents the heterojunction material composition, a2 and b2 represents the polarization charge direction, c1 and c2 represents the heterojunction band.

To date, narrow bandgap-based TJs, such as p^+^-GaN/n^+^-GaN [21], p^+^-GaN/InGaN/n^+^-GaN [22], and n^+^-GaN/p^+^-InGaN [23], have been successively reported. For wide bandgap AlGaN-based TJs, Rajan et al. first demonstrated n^+^-Al_0.3_Ga_0.7_N/In_0.25_Ga_0.75_N/p^+^-Al_0.3_Ga_0.7_N TJ UV LEDs by molecular beam epitaxy (MBE) in 2015 [24]. In 2018, a 290 nm TJ UV LED with a peak external quantum efficiency (EQE) of 2.8%, surpassing conventional LEDs, was achieved through the composition grading of AlGaN [25]. Despite the many advantages of TJ UV LEDs, challenges remain due to the limitations in p-type doping of AlGaN and the wide depletion barriers, which introduce significant drawbacks in current AlGaN-based TJs. A primary issue is the difficulty in achieving the heavy doping levels (electron and hole concentrations exceeding 10^20^ cm^−3^) required for junctions in p^+^-n^+^ TJs. To address this, a promising approach that remains unexplored is the use of short-period superlattices to replace conventional single-layer heavily doped TJs. In this study, we leverage the spontaneous and piezoelectric polarization properties inherent in group III nitride semiconductor materials, which can generate 2DEG and 2DHG within AlGaN/GaN heterojunctions. Figure 1 illustrates the theoretical structural diagrams of 2DHG and 2DEG generated by the AlGaN/GaN heterojunction structure. Here, we take GaN/AlGaN heterogeneous bonding as an example to explain the formation process of 2DEG and 2DHG.

In the Ga-plane AlGaN/GaN heterostructure, since the GaN layer below the commonly grown heterostructure is much thicker than the AlGaN layer, GaN is completely delayed and only spontaneous polarization in the direction of the C-axis [0001] exists. However, the AlGaN layer is thinner and the lattice constant of the AlGaN material is larger than that of the GaN material, so the lattice mismatch between the two causes the AlGaN layer to be subjected to tensile stress. By contrast, AlGaN experiences tensile stress in addition to spontaneous polarization. The polarization fields generated by both spontaneous and tensile stresses in AlGaN align in the same direction, opposite to the growth direction. Notably, AlGaN exhibits a higher spontaneous polarization than GaN, resulting in the appearance of polarization charge at the heterojunction interface. Here, σ_sp_ and σ_pz_ represent the surface charge densities induced by spontaneous and piezoelectric polarization, respectively. As show in Figure 1(a2), the AlGaN/GaN heterojunction interface exhibits distinct polarization charge distributions. The AlGaN layer, due to its spontaneous and piezoelectric polarization, generates a positive polarization surface charge on the left interface. Conversely, the GaN layer, influenced by its spontaneous polarization, results in a negative polarization surface charge on the right interface. The net polarization charge at the interface is a composite of these opposing charges: a significant positive charge associated with the AlGaN layer is only partially counterbalanced by a smaller negative charge associated with the GaN layer. The combined surface charge σ_sp_ + σ_pz_ (>0) is positive, resulting in a net positive polarization surface charge at the AlGaN/GaN heterojunction interface. The red line is shown in Figure 1(a2). These charges can induce electron ionization from surrounding environments, such as remote doping sources and surface states. The concentration of 2DEG generated at the heterojunction interface, even without intentional impurity doping, can reach up to approximately 10^13^ cm^−2^ [26]. In Figure 1(a3), the strong polarization electric field bends the energy band at the heterojunction interface, forming a narrow and deep conductive channel that confines the generated 2DEG.

A similar effect is observed in the GaN/AlGaN heterojunction structure. On the contrary, when the AlGaN layer at the bottom of the growing heterostructure is much thicker than the GaN layer, AlGaN is completely delayed, as shown in Figure 1(b1), and AlGaN is in a stress-free state, so only spontaneous polarization is considered. Here, the spontaneous polarization is oriented opposite to the polarization field generated by the compressive stress. At the heterojunction interface, the positive polarization charge from GaN’s spontaneous polarization and the negative polarization charge from piezoelectric polarization appear on the right interface, while the negative charge induced by AlGaN’s spontaneous polarization appears on the left interface. As shown in Figure 1(b2), AlGaN exhibits a higher spontaneous polarization than GaN, resulting in σ_sp_ + σ_pz_ (<0), which generates a net negative polarization charge at the heterojunction interface. The red line is shown in Figure 1(b2). This net negative polarization charge can induce hole ionization from the surrounding environment, such as remote doping sources and surface states. In unintentionally doped GaN/AlN heterojunctions, the concentration of 2DHG generated at the interface can reach up to 5 × 10^13^ cm^−2^ [18]. In Figure 1(b3), 2DHG is confined within a triangular channel formed by energy band bending.

Introducing Al into AlGaN alloy materials reduces the lattice constant of AlGaN, complicating Mg doping; however, using an AlGaN/GaN superlattice structure can help to address this issue. When AlGaN is epitaxially grown on a GaN film, tensile stress increases the lattice constant of AlGaN, which facilitates Mg doping [27]. Additionally, due to the difference in bandgaps between AlGaN and GaN, energy band discontinuities form at the superlattice interface, creating periodic oscillations in the energy bands. This phenomenon lowers the primary activation energy of Mg in the AlGaN barrier layer, especially at the GaN/AlGaN interface, where Mg activation energy falls below the Fermi level. As a result, ionized holes in the AlGaN barrier layer are transported to the GaN trap layer, disrupting the ionization equilibrium in the barrier layer and further promoting hole ionization. The confinement effect in the GaN trap layer aggregates holes, forming 2DHG with a much higher concentration than that produced by the heterojunction alone. This discovery suggests a pathway for tunnel junction preparation, where high doping concentrations are limited in providing sufficient hole concentrations for tunnel junctions.

In this work, we utilize short-period AlGaN/GaN superlattices in place of highly doped single-layer AlGaN as the heavily doped structure at both ends of the tunnel junction. And on this basis, we use the modeling parameters basis of DFT to perform structural modeling [28,29]. This approach allows us to study the impact of superlattice integration on the performance of UV LEDs and to explore its underlying mechanism. Based on this study, we further examine the effects of stress changes induced by introducing an intrinsic AlGaN layer within the tunnel junction on tunneling probability and current spreading. This strategy aims to maximize carrier injection efficiency, enhance internal quantum efficiency, and achieve UV LEDs with high optical output power.

## 2. Materials and Methods

**Figure 2:**
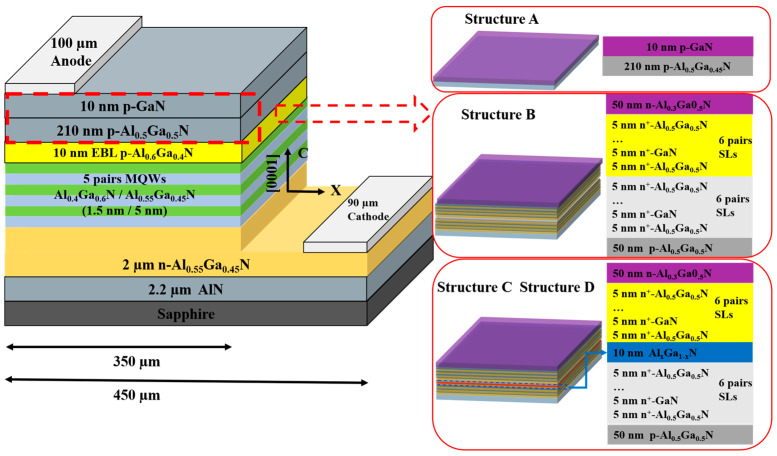
Comparison of the structures of different AlGaN-based UV-LEDs: traditional LED (Sketch and Structure A), superlattice tunnel LED (Structure B), and superlattice tunnel junction LED with dielectric AlGaN layer (Structures C and D).

The 2D AlGaN/AlGaN UV LED structure constructed using APSYS (2016 version) simulations is shown in Figure 2. Structure A adopted the conventional UV LED structural model developed by Wang [30], structure B was a superlattice tunnel junction UV LED structural model, structure C was a superlattice tunnel junction UV LED structural model with dielectric layer Al_0.4_Ga_0.6_N, and structure D was a superlattice tunnel junction UV LED structural model with dielectric layer Al_0.6_Ga_0.4_N. The three structures were identical except for the different p-type layers. The structures of the AlGaN-based UV LEDs were, from bottom to top: the c-planar sapphire substrate; the AlN buffer layer with a thickness of 2.2 µm; the n-Al_0.55_Ga_0.45_N layer with a thickness of 2 µm (n-type dopant concentration of 3 × 10^19^ cm^−3^) [31]; the multiple quantum well (MQW) stack containing 5 pairs of A_0.4_Ga_0.6_N/A1_0.55_Ga_0.45_N wells/barriers with thicknesses of 15 nm and 5 nm, respectively; and the p-Al_0.6_Ga_0.4_N EBL layer with a thickness of 10 nm (p-type doping concentration of 3 × 10^19^ cm^−3^). The p-type region of structure A with a thickness of 210 nm was the p-Al_0.5_Ga_0.5_N layer p-Al_0.5_Ga_0.5_N layer (p-type doping concentration of 3 × 10^19^ cm^−3^), and the thickness of the p-GaN layer was 10 nm (p-type doping concentration of 3 × 10^19^ cm^−3^).

Structure B used p-Al_0.5_Ga_0.5_N with a thickness of 50 nm (p-type doping concentration of 3 × 10^19^ cm^−3^); 6-cycle p^+^-Al_0.5_Ga_0.5_N/p^+^-GaN (p-type doping concentration of 1 × 10^20^ cm^−3^) with thicknesses of 5 nm in both the potential barrier and the potential well as the tunnel junction p-type layer; and 6-cycle n^+^-Al_0.5_Ga_0.5_N/n^+^-GaN (n-type doping concentration of 1 × 10^20^ cm^−3^) with both barrier and potential well thicknesses of 5 nm as the tunnel junction n-type layer.

The top layer was made of n-Al_0.3_Ga_0.7_N (n-type doping concentration of 3 × 10^19^ cm^−3^) as the ohmic contact layer.

Additionally, Al_0.4_Ga_0.6_N and Al_0.6_Ga_0.4_N with thicknesses of 10 nm were incorporated into the tunnel junctions of structures C and D, respectively, as the dielectric layers. Si and Mg were used as n-type and p-type doping sources, respectively. The electrode structure of the LED was a transverse electrode structure with the positive electrode in ohmic contact with the p-GaN and n-AlGaN contact layers, and the table size was set to 450 × 450 µm^2^.

In the quest to ascertain the fundamental factors driving carrier transport, we conducted numerical calculations employing finite element analysis. These calculations were executed utilizing the commercial software APSYS, renowned for its capability to compute the valence bands of strain-free wurtzite nitrides through a 6 × 6 k·p model [32]. During the numerical simulations, the ionization energy of the p dopant was set to 220 meV for GaN and to 470 meV for AlN [19]. The ionization energy increased linearly with the Al content of the Al_x_Ga_1−x_N alloy. The carrier transport was also sensitive to the energy band offset ratio, which was set to be 65/35 for AlGaN/AlGaN heterojunctions [33]. Based on methods developed by Fiorentini et al., the polarization charges were calculated [34]. Since the polarization charge can be screened by defect and dislocations, 50% of the theoretical polarization charges were considered in the device simulation [35]. The Shockley–Read–Hall (SRH) recombination lifetime and Auger–Meitner effect coefficient were set to be 20 ns and 1 × 10^−34^ cm^6^/s for the nonradiative recombination in MQWs, respectively. The I–V characteristic curve is influenced by the polarization charge screening coefficient, and the SRH lifetime as well as Auger–Meitner effect settings contribute to the light output power–current (L-I) characteristics. The other material parameters of nitrides adopted in the simulation can be found elsewhere [36]. Also, the tunneling process for holes and electrons was considered in our calculation by employing the transfer matrix method and the one-dimensional Schrödinger equation. Table 1 lists the specific parameters.

## 3. Results

**Figure 3:**
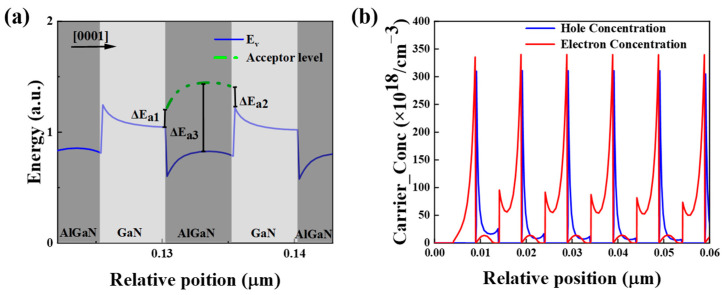
(**a**) Valence band variation and acceptor activation path, (**b**) n-type heavily doped electron concentration in the tunnel junction and p-type heavily doped hole concentration in the tunnel junction.

In order to study the change of p-type activation energy of the superlattice, we compared the activation energy of the superlattice at the barrier. In Figure 3a, we find that at the barrier AlGaN, the activation energy drops to E_a3_, and the hole excitation energy is reduced compared to the traditional p-type layer. To determine whether the carrier concentration in our designed superlattice tunnel junction met the required level for tunneling, we calculated the electron and hole concentrations induced by the superlattice structure. As shown in Figure 3b, the average electron concentration generated by the n^+^-Al_0.5_Ga_0.5_N/n^+^-GaN superlattice structure is 1.7 × 10^20^ cm^−3^. In Figure 3, the hole concentration produced by the p^+^-Al_0.5_Ga_0.5_N/p^+^-GaN superlattice structure is 3.1 × 10^20^ cm^−3^, which satisfies the carrier concentration criterion for tunneling in tunnel junctions.

**Figure 4:**
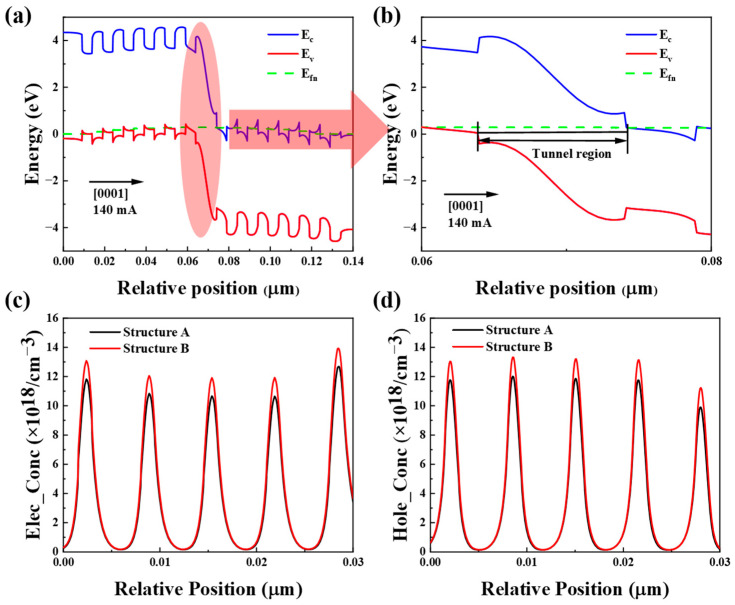
(**a**) Structure B tunnel junction band, (**b**) tunnel junction enlargement, (**c**) electron concentration and hole concentration of structure A, and (**d**) electron concentration and hole concentration structure B in MQW region at an injection current of 140 mA.

To examine the energy band structure, Fermi level (E_F_) position, and space charge region width of the designed superlattice tunnel junction, we plotted the energy band diagram of structure B, as shown in Figure 4a. The diagram reveals that the energy band structure is simple, and the E_F_ is positioned within the conduction and valence bands. In Figure 4b, the width of the space charge region is observed to be 10 nm. Driven by the electric field, electrons in the valence band of p^+^-Al_0.5_Ga_0.5_N/p^+^-GaN can tunnel through the forbidden band to the conduction band of n^+^-Al_0.5_Ga_0.5_N/n^+^-GaN, generating holes in the valence band of p^+^-Al_0.5_Ga_0.5_N/p^+^-GaN. These resulting holes drift to the active region of the LED under forward bias, enabling efficient hole injection. Furthermore, as shown in Figure 4c,d, the longitudinal distribution of electron and hole concentrations in the active region of UV LEDs with a tunnel junction structure is slightly higher than that of conventional LEDs by about 10%.

**Figure 5:**
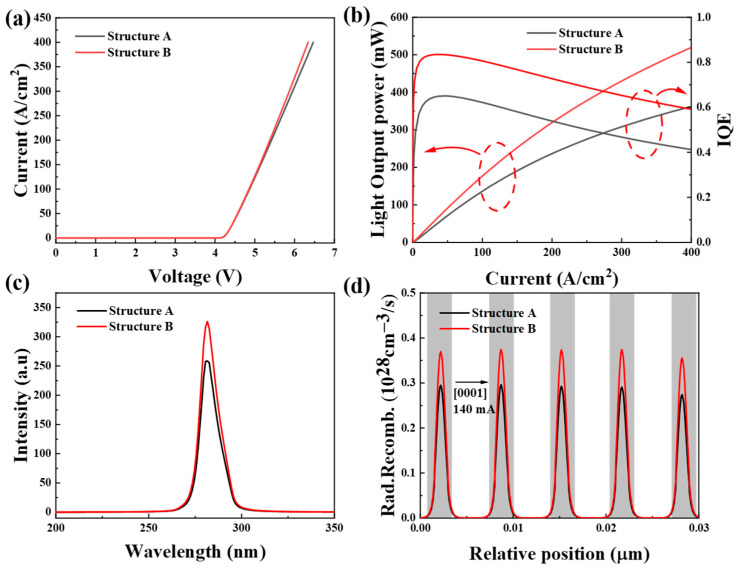
(**a**) I–V, (**b**) calculated optical power and IQE, (**c**) EL at 140 mA current at room temperature, (**d**) radiative recombination rates with structures A and B.

The I–V characteristic curves of structures A and B are presented in Figure 5a. The results indicate that the device begins to conduct at an applied voltage of approximately 4.2 V. The built-in tunnel junction operates at a reverse bias when the LED is in forward conduction, acting effectively as a resistor. Because the tunnel junction uses n-type ohmic contact, for n-type semiconductors, there may be fewer interface states and favorable electron flow, resulting in lower resistance to n-type ohmic contact. Consequently, structure A requires a slightly higher voltage to achieve the same current as structure B. Figure 5b shows the optical output power and IQE curves of structure A and structure B, respectively. The red circle represents the Y-axis corresponding to the curve. IQE can be expressed as the product of radiative recombination efficiency and carrier injection efficiency. Therefore, when we increase the tunnel junction structure, the carrier injection efficiency increases due to the increase of hole injection efficiency brought about by the local electrical site of the tunnel junction, and thus the IQE increases. Due to the increase of carrier injection efficiency, structure B shows a steeper optical output power slope, indicating a substantial increase in optical power.

When the tunnel junction is applied with reverse bias, electrons can pass through the gap band and enter the valence band to produce holes, and the tunneling current increases monotonically and rapidly with the reverse voltage, so the tunnel junction can improve the injection efficiency of holes. While the optical output power is a key indicator of LED photoelectric performance, the superlattice tunnel junction model shows obvious advantages in hole injection efficiency compared with the traditional design, which confirms its effectiveness in enhancing the luminous capacity of AlGaN-based UV LEDs. The estimated efficiency reduction of IQE depends only on the carrier injection efficiency (CIE) and the radiation recombination efficiency (RRE), while the carrier injection efficiency depends on the A, B, and C constants of the “abc” model [37]. Therefore, when the three constants are the same, we take into account the improvement of the hole injection efficiency, and structure B shows excellent internal quantum efficiency (IQE) at larger current, and the efficiency decline is reduced. Defined as (IQE_max_ − IQE_140mA_)/IQE_max_ × 100%, the decrease in the IQE of structure B is minimal. The IQE of structure A decreased from 65% to 41.2% (relative droop: 36.6%), and that of structure B decreased from 83.48% to 59.1% (relative droop: 29.2%). At low currents, the IQE is low due to the low carrier density due to the strong contribution of the Shockley–Reed–Hall recombination (related to the “a” process in the abc model)

Figure 5c shows the room-temperature EL intensity for both structures at 140 mA, with both exhibiting the same peak emission wavelength. The EL strength of structure B is over 20% higher than that of structure A. Figure 5d shows the radiative recombination rate in the active region, where the improved injection efficiency of structure B leads to an increase in the carrier concentration in the active region, so the radiative rate is higher than that of structure A.

The polarized tunnel junction is a kind of p^+^-i-n^+^ heterojunction formed on the basis of the traditional p^+^-n^+^ homogenous junction. Due to the existence of spontaneous and piezoelectric polarization in the p^+^-i-n^+^ heterojunction, polarization can induce high density polarization surface charges at the p^+^-i and i-n^+^ interfaces. When the i layer is subjected to compressive stress, the p^+^-i interface will form a negative polarization surface charge, and the i-n^+^ interface will form a positive polarization surface charge. When the i layer is subjected to tensile stress, the p^+^-i interface will form a positive polarization surface charge, and the i-n^+^ interface will form a negative polarization surface charge. AlGaN with Al groups 0.4 and 0.6 are added to the tunnel junction.

**Figure 6:**
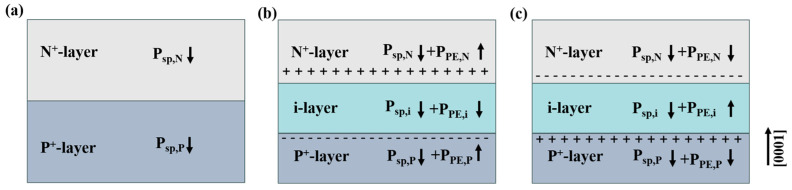
Direction of polarization field strength at tunnel junction, (**a**) conventional tunnel junction, (**b**) compressive stress tunnel junction, (**c**) tensile stress tunnel junction.

**Figure 7:**
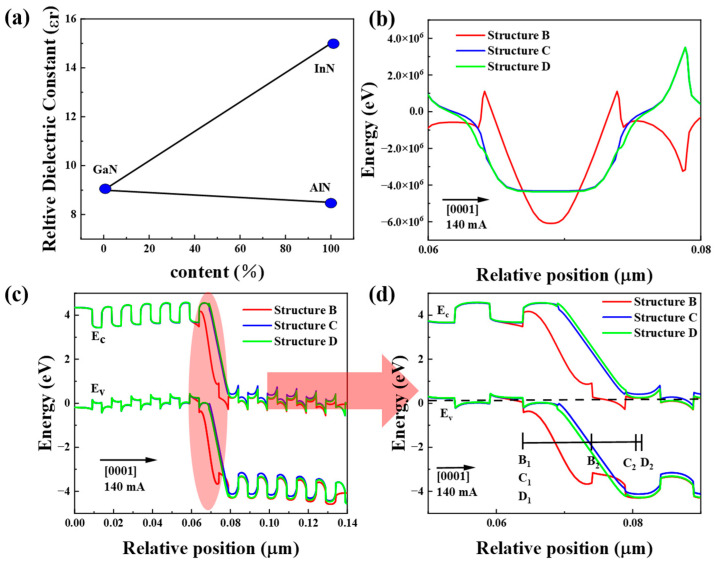
(**a**) Relationships between relative dielectric constants and In content in InGaN and Al content in AlGaN, (**b**) electric field distributions of tunnel junctions B, C, and D, (**c**) structure B, C, and D’s tunnel junctions band, (**d**) tunnel junction enlargement.

Then, we further studied its induced spontaneous polarization and the effect of piezoelectric polarization on the stress change of the dielectric layer, as shown in Figure 6. Due to the spontaneous and piezoelectric polarization of the p^+^-i-n^+^ heterojunction, the stress of the traditional tunnel junction comes from the spontaneously polarized P_sp,N_ and P_sp,P_, while the stress of the polarized tunnel junction comes not only from the spontaneous polarization but also from the piezoelectric polarization of the i layer under the action of compressive or tensile stress P_PE,i_. Therefore, when layer i is subjected to compressive stress, the spontaneous polarization of layer n, layer i, and layer p is opposite to the growth direction, the piezoelectric polarization of layer n and layer p is the same as the growth direction, and the piezoelectric polarization of layer i is opposite to the growth direction. Therefore, when the i layer is subjected to tensile stress, the piezoelectric polarization of the n layer and the p layer is opposite to the growth direction, and the piezoelectric polarization of the i layer is the same to the growth direction. Due to the difference in stress, the coupled electric field strength of the tunnel junction is also affected. The coupled electric field strength E of the polarized tunnel junction is calculated as follows [30]:

(1) $$E=q\times \left|\frac{N_{\mathrm{dopant}}\times L_{\mathrm{dopant}}\pm \sigma_{\rho }}{\varepsilon_{r}\times \varepsilon_{0}}\right|$$

where E is the tunnel junction field strength intensity, q is the unit electron charge, ε_0_ is the absolute permittivity, ε_r_ is the average relative permittivity of the polarized tunnel junction, N_dopant_ is the concentration of ionized impurities in the space charge, L_dopant_ is the depletion layer width, and σ_p_ is the polarization-induced surface charge density. The symbol ± is the direction of E_p_. Therefore, when ε_r_ is smaller, the tunnel junction field strength intensity is higher. According to the relationship curve of ε_r_ with the change in the In component in InGaN and the Al component in AlGaN, as shown in Figure 7a, when ε_r_ decreases with the increase in the Al component, ε_r_ increases with the increase in the In component. Therefore, according to the tunnel junction formula, the tunneling probability of the tunnel junction increases with the increase of the tunnel barrier height and the depletion layer width, then the electron tunneling probability of the tunnel junction is expressed as follows [31]:

(2) $$T_{i}=\exp\left(-\frac{4}{3}\sqrt{\frac{2m^{*}\phi_{b}}{\hbar^{2}e}}w_{d}\right)$$

where ϕ_b_, w_d_, m*, ħ, and e are the tunnel barrier height, depletion width, effective mass, reduced Planck’s constant, and electron charge, respectively, where m* can be calculated by calculating the folded effective mass of electrons and holes. m* (GaN) = 0.18, m* (AlN) = 0.27 [38], approximates the effective mass of the electron.

In order to change the band structure of the tunnel junction and make it have a higher barrier and depletion layer width, AlGaN was selected as dielectric layer material.

As shown in Figure 7b, the E_c_ strengths of the three tunnel junction structures are shown, and the peak values of E_c_ are calculated to obtain E_peak_ (structure B) = 6.1 × 10^6^ V/cm, E_peak_ (structure C) = 4.3 × 10^6^ V/cm, and E_peak_ (structure D) = 4.2 × 10^6^ V/cm, and the average field strengths of E_c_ are calculated by integrating the E_c_ throughout the tunnel junction to obtain $\bar{E}$ (structure B) = 2 × 10^6^ V/cm, $\bar{E}$ (structure C) = 2.2 × 10^6^ V/cm, and $\bar{E}$ (structure D) = 2.4 × 10^6^ V/cm. As shown in the energy band diagrams of the three tunneling junctions in Figure 7c, the electrons in the valence band of p^+^-Al_0.5_Ga_0.5_N/p^+^-GaN are to pass through the forbidden bands and go into the conduction band of n^+^-Al_0.5_Ga_0.5_N/ n^+^-GaN under the driving of the electric field. At the same time, it has the same effect on structures B and C. As in Figure 7d, we can observe three tunnel junction widths: |B_1_B_2_| = 10 nm; |C_1_C_2_| = 18 nm; and | D_1_D_2_| = 20 nm. For the triangular barriers, the relationship between the average forbidden bandwidth (${\bar{E}}_{g}$), the average electric field strength ($\bar{E}$), as well as the width of the tunnel junction (W) is expressed as follows:

(3) $${\bar{E}}_{g}=q\bar{E}\times W$$

According to Figure 7d, we can observe that structure D has a higher barrier height and depletion layer width compared with the other two types. Calculations yield ${\bar{E}}_{g}$ structure D) > ${\bar{E}}_{g}$ (structure C) > ${\bar{E}}_{g}$ (structure B). The tunneling probabilities of structures B, C, and D are calculated to be P_0_ (structure D) > P_0_ (structure C) P_0_ (structure B), and thus structure D has the highest tunneling probability. This yields the highest hole injection efficiency.

**Figure 8:**
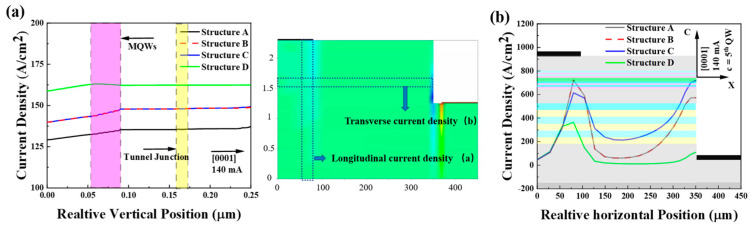
Longitudinal and lateral distributions of current densities in MQWs of LEDs with structures A, B, C, and D, (**a**) current densities along growth direction at the center of p-metal x = 75 μm, c = 0 μm to 250 μm, (**b**) in-plane current density distribution c = 5th QW, x = 0 to 350 μm.

Figure 8a shows the longitudinal distribution of current density in the MGW of structures A, B, C, and D (with respect to the mesa edge x = 75 μm, c = 0 μm to 250 μm), while Figure 8b compares the transverse current density distribution in the fifth quantum well (with respect to the mesa edge c = 5th QW, x = 0 to 350 μm). For LEDs with a lateral structure, the tunnel junction enhances current spreading, as shown in Figure 8a, where structure D exhibits the highest longitudinal current density. Because of the transverse distribution of current, structure D has better current expansion because the current accumulates less at the p-metal edge, while in other structures, the current accumulates at the p-metal edge, so the central part of the p-metal receives less current.

In Figure 8b, all four structures display an uneven current distribution due to the lateral structure, with higher current density from 0 μm to 125 μm and lower density from 125 μm to 300 μm. However, in structure A, due to homogeneous resistance, current preferentially flows into the MQW along the shortest conductive path, leading to a highly uneven lateral current distribution in the MQW. By contrast, tunnel junctions B, C, and D comprise multilayer materials with varying conductivities, which create transverse conductive channels at material interfaces, thereby enhancing the lateral uniformity of current density.

The current spreading capability depends on the tunnel junction resistance, which in turn is influenced by the Al content in AlGaN. Among the structures, the polarized tunnel junction in structure D has the highest tunnel junction resistance due to its higher Al component. This characteristic not only improves electron tunneling probability and hole injection efficiency but also increases tunnel junction resistance, which mitigates current crowding, enhances transverse current spreading, and improves LED emission uniformity. Moreover, due to the change in band structure and carrier transmission path, the current expansion is better.

**Figure 9:**
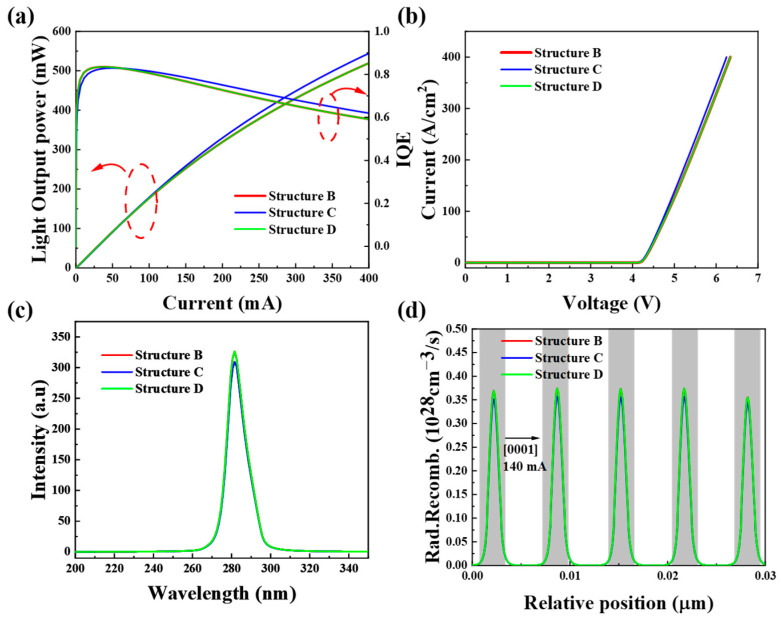
(**a**) Calculated optical power and IQE, (**b**) I–V, (**c**) EL at 140 mA current at room temperature, (**d**) radiative recombination rates with structures B, C, and D.

To evaluate the UV LED performance of the three tunnel junction structures, we first compared their IQE and light output power curves, as shown in Figure 9a. The red circle is the curve corresponding to the Y-axis. The results indicate that structure D has the highest IQE, structure C exhibits the highest light output power, and structure D shows the largest efficiency droop rate at 28.9%. This may be due to the higher tunneling junction resistance in structure D, which exacerbates the reduction in optical output power and IQE at high currents.

Figure 9b compares the I–V characteristic curves of the three structures, revealing that structure C achieves the highest current at a given voltage, likely due to its lower tunnel junction resistance. Figure 9c presents the electroluminescence (EL) spectra of the three structures, all of which emit at a wavelength of 280 nm, with structure D exhibiting the highest luminescence intensity. Finally, Figure 9d shows the radiative recombination rate in the active region for each structure. The grey background represents the quantum well region. Structure D achieves the highest radiative recombination rate, attributed to its higher tunneling probability and increased hole concentration in the active region.

## 4. Discussion

The use of the superlattice tunneling junction and the introduction of the dielectric layer are beneficial to improving the IQE and optical output power of UV LEDs, which is mainly due to the tunneling junction improving the hole injection efficiency, while the dielectric layer optimizes the current expansion due to the change in internal resistance when the Al component is high and further improves the optoelectronic performance when the Al component is low through the change in stress. Moreover, according to the performance results, structure D has a high tunneling probability, so the peak value of the IQE is slightly higher than that of other structures. However, due to its high internal resistance, the SRH compound increases, which exacerbates the decline in the IQE and the decline in optical output power under high current density. On this basis, compared with other UV LEDs, the optical output power is greatly improved compared with that developed by Wang at the same operational wavelength. Further, these improvements are closely related to the unique properties of group III nitride materials. In particular, the polarization characteristics and band structure of AlGaN-based materials are crucial for carrier injection and recombination processes. In AlGaN with a high aluminum composition, built-in polarized electric fields help to enhance carrier injection, and the introduction of dielectric layers further adjusts these electric fields and optimizes carrier transport and distribution. In addition, the high electron affinity and direct band gap properties of group III nitrides make them highly efficient in the UV spectral range. Our study highlights that fine-tuning of UV LED performance can be achieved by precisely controlling the composition and stress of these group III nitride materials, which has important implications for advancing the development of high-performance UV optoelectronic devices.

## 5. Conclusions

In this work, UV LEDs with a peak emission wavelength of 280 nm were systematically analyzed using APSYS commercial software, focusing on the impact of incorporating a superlattice tunnel junction on hole injection. By comparing the performance of the superlattice tunnel junction structure with that of a conventional LED, it was found that the superlattice tunnel junction significantly enhances both the optical output power and IQE of the LED. Additionally, a dielectric layer was introduced into the superlattice tunnel junction to examine its effects on tunneling probability under compressive and tensile stresses.

Furthermore, by evaluating the current spreading capability and tunneling resistance across three tunnel junction configurations, it was observed that the dielectric layer with composition Al_0.6_Ga_0.4_N exhibited the highest tunneling resistance and the best current spreading performance. Comparative analysis showed that Al_0.4_Ga_0.6_N as the dielectric layer yielded the highest optical output power, while Al_0.6_Ga_0.4_N provided the highest IQE. Therefore, the UV LED structure and device properties proposed in this study show significant potential for advancing future research on tunnel junction-based UV LEDs.

## Figures and Tables

**Table 1:** Model parameters used in the simulation.

Energy band offset ratio	65/35
Theoretical polarization charges	50%
The Shockley–Read–Hall (SRH) recombination lifetime	20 ns
Auger–Meitner effect coefficient	1 × 10^−34^ cm^6^/s

## Data Availability

All data that support the findings of this study are included within the article.

## References

[1] Sun H.D., Mitra S., Subedi R.C., Zhang Y., Guo W., Ye J.C., Shakfa M.K., Ng T.K., Ooi B.S., Roqan I.S. (2019). Unambiguously Enhanced Ultraviolet Luminescence of AlGaN Wavy Quantum Well Structures Grown on Large Misoriented Sapphire Substrate. Adv. Funct. Mater..

[2] Yu H., Memon M.H., Wang D., Ren Z., Zhang H., Huang C., Tian M., Sun H., Long S. (2021). AlGaN-based deep ultraviolet micro-LED emitting at 275 nm. Opt. Lett..

[3] Li D.B., Jiang K., Sun X.J., Guo C.L. (2018). AlGaN Photonics: Recent Advances in Materials and Ultraviolet Devices. Adv. Opt. Photonics.

[4] Khan A., Balakrishnan K., Katona T. (2008). Ultraviolet Light-emitting Diodes based on Group Three Nitrides. Nat. Photonics.

[5] He L.F., Zhang K., Wu H.L., He C.G., Zhao W., Wang Q., Li S.T., Chen Z.T. (2021). Efficient Carrier Transport for 368 nm Ultraviolet LEDs with A p-AlInGaN/AlGaN Short-Period Superlattice Electron Blocking Layer. J. Mater. Chem. C.

[6] Takano T., Mino T., Sakai J., Noguchi N., Tsubaki K., Hirayama H. (2017). Deep-Ultraviolet Light-emitting Diodes with External Quantum Efficiency Higher than 20% at 275 nm Achieved by Improving Light-extraction Efficiency. Appl. Phys. Express.

[7] Inoue S.I., Tamari N., Taniguchi M. (2017). 150 mW Deep-ultraviolet Light-emitting Diodes with Large-area AlN Nanophotonic Light-extraction Structure Emitting at 265 nm. Appl. Phys. Lett.

[8] Kojima K., Nagasawa Y., Hirano A., Ippommatsu M., Honda Y., Amano H., Akasaki I., Chichibu S.F. (2019). Carrier Localization Structure Combined with Current Micropaths in AlGaN Quantum Wells Grown on An AIN Template with Macrosteps. Appl. Phys. Lett..

[9] Kneissl M., Seong T.Y., Han J., Amano H. (2019). The Emergence and Prospects of Aeep-ultraviolet Light-emitting Diode Technologies. Nat. Photonics.

[10] Yu C.T., Lai W.C., Yen C.H., Chang S.J. (2014). Effects of InGaN Layer Thickness of AlGaN/InGaN Superlattice Electron Blocking Layer on the Overall Efficiency and Efficiency Droops of GaN-based Light Emitting Diodes. Opt. Express.

[11] Chu C.S., Tian K.K., Che J.M., Shao H., Kou J.Q., Zhang Y.H., Li Y., Wang M.Y., Zhu Y.H., Zhang Z.H. (2019). On the Origin of Enhanced Hole Injection for AlGaN-based Deep Ultraviolet Light-Emitting Diodes with AlN Insertion Layer in p-Electron Blocking Layer. Opt. Express.

[12] Zhang Z.H., Chen S.W.H., Zhang Y.H., Li L.P., Wang S.W., Tian K.K., Chu C.S., Fang M.Q., Kuo H.C., Bi W.G. (2017). Hole Transport Manipulation To Improve the Hole Injection for Deep Ultraviolet Light-Emitting Diodes. ACS Photonics.

[13] Zhao Z., Liu Y., Li P.X., Zhou X.W., Yang B., Xiang Y.R. (2024). Enhanced Hole Injection in AlGaN-Based Ga-Polar Ultraviolet Light-Emitting Diodes with Polarized Electric-Field Reservoir Electron Barrier. Micromachines.

[14] Lang J., Xu F.J., Ge W.K., Liu B.Y., Zhang N., Sun Y.H., Wang J.M., Wang M.X., Xie N., Fang X.Z. (2019). Greatly Enhanced Performance of AlGaN-based Deep Ultraviolet Light Emitting Diodes by Introducing A Polarization Modulated Electron Blocking Layer. Opt. Express.

[15] Jeon S.R., Song Y.H., Jang H.J., Yang G.M., Hwang S.W., Son S.J. (2001). Lateral Current Spreading in GaN-based Light-emitting Diodes Utilizing Tunnel Contact Junctions. Appl. Phys. Lett..

[16] Jeon S.R., Cho M.S., Yu M.A., Yang G.M. (2002). GaN-based Light-emitting Diodes Using Tunnel Junctions. IEEE J. Sel. Top. Quant..

[17] Lee C.M., Chuo C.C., Chen I.L., Chang J.C., Chyi J.I. (2003). High-brightness inverted InGaN-GaN multiple-quantum-well light-emitting diodes without a transparent conductive layer. IEEE Electron Device Lett..

[18] Chaudhuri R., Bader S.J., Chen Z., Muller D.A., Xing H.G., Jena D. (2019). A Polarization-induced 2D Hole Gas in Undoped Gallium Nitride Quantum Wells. Science.

[19] Simon J., Protasenko V., Lian C.X., Xing H.L., Jena D. (2010). Polarization-Induced Hole Doping in Wide-Band-Gap Uniaxial Semiconductor Heterostructures. Science.

[20] Esaki L. (1958). New Phenomenon in Narrow Germanium p-n Junctions. Phys. Rev..

[21] Neugebauer S., Hoffmann M.P., Witte H., Bläsing J., Dadgar A., Strittmatter A., Niermann T., Narodovitch M., Lehmann M. (2017). All Metalorganic Chemical Vapor Phase Epitaxy of p/n-GaN Tunnel Junction for Blue Light Emitting Diode Applications. Appl. Phys. Lett..

[22] Zhang Z.H., Tan S.T., Kyaw Z., Ji Y., Liu W., Ju Z.G., Hasanov N., Sun X.W., Demir H.V. (2013). InGaN/GaN Light-emitting Diode with a Polarization Tunnel Junction. Appl. Phys. Lett..

[23] Takeuchi T., Hasnain G., Corzine S., Hueschen M., Schneider R.P., Kocot C., Blomqvist M., Chang Y.L., Lefforge D., Krames M.R. (2001). GaN-based Light Emitting Diodes with Tunnel Junctions. JPN J. Appl. Phys..

[24] Zhang Y.W., Krishnamoorthy S., Johnson J.M., Akyol F., Allerman A., Moseley M.W., Armstrong A., Hwang J., Rajan S. (2015). Interband Tunneling for Hole Injection in III-nitride Ultraviolet Emitters. Appl. Phys. Lett..

[25] Zhang Y.W., Jamal-Eddine Z., Akyol F., Bajaj S., Johnson J.M., Calderon G., Allerman A.A., Moseley M.W., Armstrong A.M., Hwang J. (2018). Tunnel-injected Sub 290 nm uUtra-violet Light Emitting Diodes with 2.8% External Quantum Efficiency. Appl. Phys. Lett..

[26] Ibbetson J.P., Fini P.T., Ness K.D., DenBaars S.P., Speck J.S., Mishra U.K. (2000). Polarization Effects, Surface States, and the Source of Electrons in AlGaN/GaN Heterostructure Field Effect Transistors. Appl. Phys. Lett..

[27] Wang X., Wang W., Wang J.L., Wu H., Liu C. (2017). Experimental Evidences for Reducing Mg Activation Energy in High Al-content AlGaN Alloy by MgGa δ Doping in (AlN)m/(GaN)n Superlattice. Sci. Rep..

[28] Alves Machado Filho M., Hsiao C.-L., Dos Santos R.B., Hultman L., Birch J., Gueorguiev G.K. (2023). Self-Induced Core-Shell InAlN Nanorods: Formation and Stability Unraveled by Ab Initio Simulations. ACS Nanosci. Au.

[29] Machado M.A., Farmer W., Hsiao C.L., dos Santos R.B., Hultman L., Birch J., Ankit K., Gueorguiev G.K. (2024). Density Functional Theory-Fed Phase Field Model for Semiconductor Nanostructures: The Case of Self-Induced Core-Shell InAlN Nanorods. Cryst. Growth Des..

[30] Wang Y., Zhang Z.H., Guo L., Chen Y.X., Li Y.H., Qi Z.B., Ben J.W., Sun X.J., Li D.B. (2021). Calculating the Effect of AlGaN Dielectric Layers in a Polarization Tunnel Junction on the Performance of AlGaN-Based Deep-Ultraviolet Light-Emitting Diodes. Nanomaterials.

[31] Deng G.Q., Yu J.Q., Niu Y.F., Zhang L.D., Ma H.T., Wang Y.S., Zuo C.C., Yang S.X., Han X., Chen L. (2024). Demonstration of Full AlGaN Tunnel Junction Ultraviolet LED. ACS Photonics.

[32] Chuang S.L., Chang C.K. (1996). k·p method for strained wurtzite semiconductors. Phys. Rev. B.

[33] Piprek J. (2014). Origin of InGaN/GaN Light-emitting Diode Efficiency Improvements Using Tunnel-Junction-cascaded Active Regions. Appl. Phys. Lett..

[34] Fiorentini V., Bernardini F., Ambacher O. (2002). Evidence for Nonlinear Macroscopic Polarization in III-V Nitride Alloy Heterostructures. Appl. Phys. Lett..

[35] Renner F., Kiesel P., Döhler G.H., Kneissl M., Van de Walle C.G., Johnson N.M. (2002). Quantitative Analysis of the Polarization Fields and Absorption Changes in InGaN/GaN Quantum Wells with Electroabsorption Spectroscopy. Appl. Phys. Lett..

[36] Vurgaftman I., Meyer J.R. (2003). Band Parameters for Nitrogen-containing Semiconductors. J. Appl. Phys..

[37] Xu R.Q., Kang Q.S., Zhang Y.W., Zhang X.L., Zhang Z.H. (2023). Research Progress of AlGaN-Based Deep Ultraviolet Light-Emitting Diodes. Micromachines.

[38] Suzuki M., Uenoyama T., Yanase A. (1995). First-principles Calculations of Effective-mass Parameters of AlN and GaN. Phys. Rev. B.

